# Recent advances in graphene-based electroanalytical devices for healthcare applications

**DOI:** 10.1039/d3nr06137j

**Published:** 2024-05-29

**Authors:** Vinay Kammarchedu, Heshmat Asgharian, Keren Zhou, Pouya Soltan Khamsi, Aida Ebrahimi

**Affiliations:** a Department of Electrical Engineering, The Pennsylvania State University University Park Pennsylvania 16802 USA sue66@psu.edu; b Center for Atomically Thin Multifunctional Coatings, The Pennsylvania State University University Park Pennsylvania 16802 USA; c Materials Research Institute, The Pennsylvania State University University Park Pennsylvania 16802 USA; d Department of Biomedical Engineering, The Pennsylvania State University University Park Pennsylvania 16802 USA

## Abstract

Graphene, with its outstanding mechanical, electrical, and biocompatible properties, stands out as an emerging nanomaterial for healthcare applications, especially in building electroanalytical biodevices. With the rising prevalence of chronic diseases and infectious diseases, such as the COVID-19 pandemic, the demand for point-of-care testing and remote patient monitoring has never been greater. Owing to their portability, ease of manufacturing, scalability, and rapid and sensitive response, electroanalytical devices excel in these settings for improved healthcare accessibility, especially in resource-limited settings. The development of different synthesis methods yielding large-scale graphene and its derivatives with controllable properties, compatible with device manufacturing – from lithography to various printing methods – and tunable electrical, chemical, and electrochemical properties make it an attractive candidate for electroanalytical devices. This review article sheds light on how graphene-based devices can be transformative in addressing pressing healthcare needs, ranging from the fundamental understanding of biology in *in vivo* and *ex vivo* studies to early disease detection and management using *in vitro* assays and wearable devices. In particular, the article provides a special focus on (i) synthesis and functionalization techniques, emphasizing their suitability for scalable integration into devices, (ii) various transduction methods to design diverse electroanalytical device architectures, (iii) a myriad of applications using devices based on graphene, its derivatives, and hybrids with other nanomaterials, and (iv) emerging technologies at the intersection of device engineering and advanced data analytics. Finally, some of the major hurdles that graphene biodevices face for translation into clinical applications are discussed.

## Abstract 2

Recent advances in graphene-based electroanalytical biodevices: different methods for graphene synthesis, functionalization, device fabrication, and transduction mechanisms are discussed for various healthcare applications.

## Graphical abstract

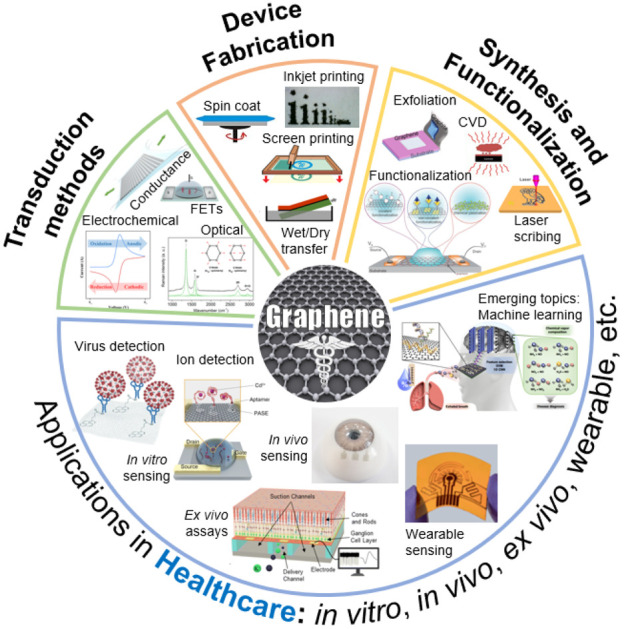

## Introduction

Graphene is a two-dimensional (2D) sheet of hexagonal sp^2^-bonded carbon,^1^ wherein each carbon atom is bonded to three neighbouring atoms, forming a highly stable lattice.^2^ This arrangement grants graphene exceptional mechanical strength.^3^ Moreover, its 2D nature and high surface area-to-volume ratio offer an excellent platform for chemical interactions, making graphene an excellent material for sensing applications.^4^ The hallmark of graphene's electrical properties lies in its remarkable electron mobility, surpassing that of traditional semiconductors.^5^ This property, coupled with its high (and tunable) electrical conductivity, positions graphene as an ideal candidate for constructing advanced electrical and electrochemical devices. Interestingly, drastic changes to the electronic band structure are observed when the thickness of graphene increases from monolayer to a few layers, and further to thick graphitic crystals.^6–8^ Thus, it is critical to elucidate these differences when comparing the sensing performance of devices. In this review article, we use ISO standard terminology^9^ to refer to graphene, *i.e.*, the monolayer as graphene, and the subsequent thicknesses with appended layer numbers (*e.g.*, 2LG, 3LG, and FLG for 4–10 layers). Furthermore, for brevity in this paper, the abbreviation GR is used to refer to graphene up to 10 layers in thickness.

**Fig. 1:**
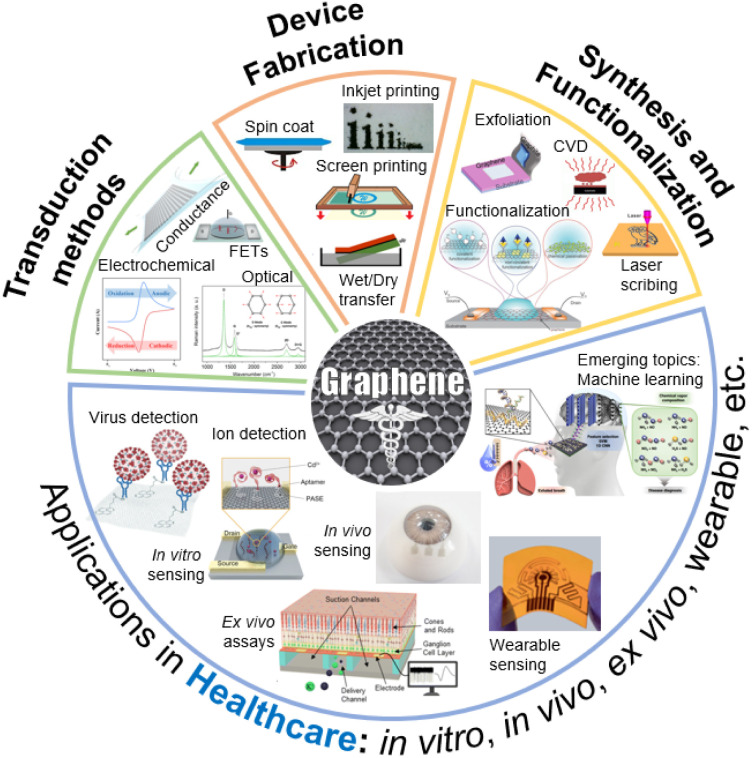
A snapshot of the scope of this review. Different ways to synthesize graphene, functionalization, device fabrication, and sensor transduction mechanisms are discussed. Adapted with permission from ref. 16 and 17. Copyright 2018–2022 American Chemical Society. Adapted with permission from ref. 18–20. Copyright 2020–2021 John Wiley and Sons. Adapted with permission from ref. 21–23. Copyright 2020 Springer Nature. Adapted with permission from ref. 24. Copyright 2020 MDPI. Adapted with permission from ref. 25–27. Copyright 2022 The Royal Society of Chemistry.

Fig. 1 provides a summary of the scope of this review article. The first section discusses various synthesis methods and their suitability for building biodevices in healthcare applications. Central to the successful integration of GR into electroanalytical devices is the development of precise and scalable synthesis techniques.^10^ Various top-down and bottom-up methods enable the synthesis of GR with vast differences in defect densities, surface functional groups, hetero-atom presence, *etc*. The synthesis techniques also produce GR of various dimensions.^11^ For example, quantum dots are zero dimensional, whereas graphene nanoribbons are one dimensional. A highly three dimensional and porous GR can also be produced using techniques such as laser scribing.^12^ Furthermore, incorporating functional groups onto the GR surface is a pivotal step in tailoring its interaction with target bioanalytes.^13^ GR's basal plane offers a plethora of π-conjugated electrons,^14^ while its edges and defects can act as active sites for chemical interactions.^15^

The versatility of the physiochemical properties of GR enables the design and manufacturing of diverse electroanalytical device architectures, as highlighted in the Device Manufacturing section. In this section, various device fabrication methods along with specific examples of devices are described. The following section focuses on the transduction mechanisms, including field-effect transistors, electrochemical methods, and electro-optical devices, and their respective uses in healthcare settings. The next section of this article focuses on emerging healthcare applications. GR-based devices, characterized by their ability to precisely analyze biological samples, detect biomarkers, and monitor physiological parameters, hold the potential to revolutionize disease diagnosis, personalized medicine, remote patient monitoring, and life sciences research. As technology advances, the integration of electroanalytical devices with digital platforms holds the promise of ushering in an era of preventive and precision medicine, ultimately improving patient outcomes and healthcare management on a global scale.

Finally, the current progress and the existing challenges involved in translating GR-based biodevices into practical setting are discussed. While the potential of GR-based electroanalytical devices in healthcare is undeniable, several challenges remain to be addressed.^28^ Achieving reproducibility in GR synthesis and scalability of device preparation (with high yield) are crucial for the widespread adoption of these devices. The functionalization processes require careful consideration to maintain GR's properties while enhancing the device performance.^29^ Moreover, the integration of GR-based sensors into complex biological matrices, especially for *in vivo* analysis and long-term readout demands robustness, stability, and biocompatibility. As the global healthcare industry faces hurdles ranging from the early detection of diseases to the management of chronic conditions, we hope that this article sheds some light on the transformative role that GR-based devices can play in addressing such critical needs.

## Synthesis and functionalization

**Fig. 2:**
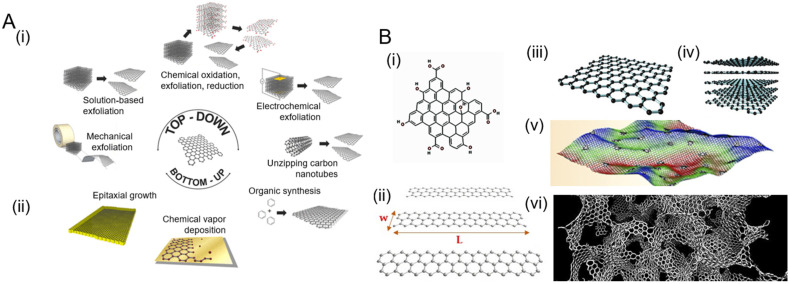
Various routes for graphene synthesis and the different dimensions of graphene that are produced. [A] Schematic showing common synthesis approaches: (i) top-down methods create graphene by removing material, whereas (ii) bottom-up methods produce graphene by sequential addition of suitable atoms. Adapted with permission from ref. 32. Copyright 2014 American Chemical Society. [B] Different dimensions of graphene: (i) quantum dots and (ii) nanoribbons. Adapted with permission from ref. 33. Copyright 2018 MDPI. (iii) Graphene sheets, (iv) few layer graphene (FLH), and (v) defective and corrugated graphene. Adapted with permission from ref. 34. Copyright 2021 American Chemical Society. (vi) 3D graphene obtained by the combination of graphene sheets of various thickness and defects. Adapted with permission from ref. 35. Copyright Qin *et al.*

Synthesis techniques and further processing steps greatly influence the properties and quality of the GR that is produced.^30,31^ In this section, we first provide an overview of different dimensionalities of graphene that have been explored in biodevices. Next, we discuss the synthesis methods (Fig. 2A), which can be broadly categorized as top-down (Fig. 2A(i)) or bottom-up (Fig. 2A(ii)) approaches depending on whether GR is produced by removing nanostructures from the bulk solid or adding suitable atoms, respectively. We then summarize the various processing/functionalization methods that further transform the physiochemical properties of GR.

### Dimensions of graphene

The basic two-dimensional graphene (2D-GR) building block can be used to visualize the creation of various carbon-based materials by tailoring, folding, and stacking graphene to create materials from zero to three dimensions (Fig. 2B). Zero-dimensional graphene quantum dots (0D-GR) are nanometer-sized particles (Fig. 2B(i)), such as nanocrystals and buckyballs. They are usually rich in functional groups such as hydroxyl, epoxy, and carboxyl on their edges.^36^ 0D-GR have interesting electrical and chemical properties that can be tuned by the modification of their dimensions and surface chemistry, especially for the observation of optical phenomena.^37^ Common routes for the synthesis of 0D-GR^38^ include plasma etching, liquid exfoliation, hydrothermal, and solvothermal methods.^39^ One-dimensional graphene (1D-GR) is produced by the confinement of GR in a certain direction (Fig. 2B(ii)).^40^ Nanoribbons of graphene can be produced by top-down methods such as patterning and etching, catalytic cutting, and *in situ* lithography.^41^ The electrical and optical properties of 1D-GR can be tuned by doping, strain engineering, and modifying chemical functional groups at the edge.^42^ 2D-GR (Fig. 2B(iii)) can be stacked to form FLG (Fig. 2B(iv)). Three-dimensional graphene (3D-GR) such as graphene foams, hydrogels, and aerogels comprised of defective and corrugated graphene (Fig. 2B(v)) enables a porous and high specific surface area (Fig. 2B(vi)). 3D-GR is especially promising for energy storage, conversion, catalysis, and sensing applications. Synthesis methods for 3D-GR include hydrothermal, chemical vapor deposition, and on-site polymerization techniques.^43^ The properties of 3D-GR can be tuned by doping, surface chemical functionalization, defect engineering, and porosity engineering.^43^

### Top-down synthesis strategies

The top-down strategy for GR production generally entails the separation of material from carbon sources such as graphite crystals and powders (Fig. 2A(i)). These methods destructively attempt to separate the existing stacked layers of carbon to obtain monolayer graphene to FLG. One of the very first top-down methods to isolate graphene from graphite was based on micromechanical exfoliation using a tape technique.^44^ Even though it is capable of producing high quality graphene, this method does not yield very large area graphene and is unsuitable for large scale production and commercialization. In contrast, liquid phase exfoliation (LPE) is used to exfoliate graphite using thermal, chemical, or mechanical processes on a large scale.^45^ Electrochemical exfoliation can also be used to exfoliate graphite by intercalating ionic species into graphite where they form gaseous molecules, expanding and exfoliating graphite into GR sheets.^46^ Graphite can also be oxidized chemically and subsequently reduced to form GR such as in Hummers’ method^47^ and the Brodie method.^31^ For a thorough review of different top-down methods for the synthesis of GR, we refer the readers to a review article by Kumar *et al.*^48^

### Bottom-up synthesis strategies

Bottom-up strategies focus on building layers of graphene using suitable precursors (Fig. 2A(ii)). These methods may include chemical vapor deposition (CVD),^49^ epitaxial growth,^50^ and thermal pyrolysis, among others.^51^ CVD is a popular method to produce large area 2D monolayer to FLG films. Many hydrocarbon precursors and a variety of substrates (*e.g.*, copper) have been used to grow these graphene films.^52^ The main drawback of the CVD process is its sensitivity to the growth parameters and transfer methods that limit its overall quality in devices as well as requiring high temperatures. Epitaxial growth of graphene is also possible on silicon carbide substrates.^53^ Thermal pyrolysis^54^ or laser-based scribing of various carbon-containing sources (which are discussed later) also yield GR with diverse structures and properties.^12^ These methods generally result in GR that has a higher density of defects due to the chaotic formation process during manufacturing.^16^ For a thorough review of different top-down methods for the synthesis of GR, we refer the readers to a review article by Gutiérrez-Cruz *et al.*^55^

### Functionalization of graphene

**Fig. 3:**
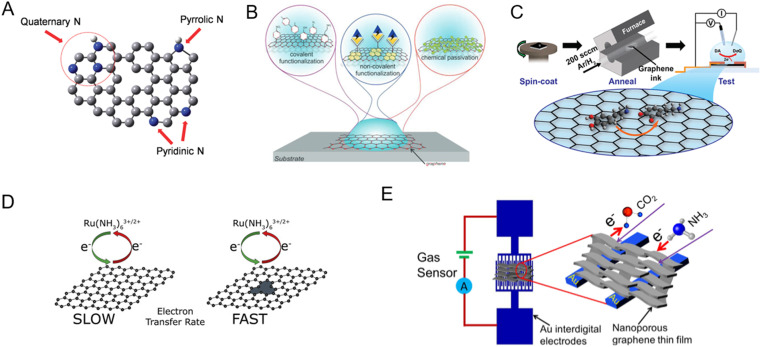
Different modification methods to tune the sensitivity and specificity of graphene to target analytes. [A] Doping with heteroatoms (example: nitrogen-doped graphene was shown to enhance the electrocatalytic activity of hydrogen peroxide reduction, which was then utilized to detect glucose). Adapted with permission from ref. 61. Copyright 2014 American Chemical Society. [B] Surface chemical modification *via* covalent, non-covalent, and chemical passivation. Adapted with permission from ref. 18. Copyright 2020 John Wiley and Sons. [C] Annealing (example: ink-based electrochemical sensors; tuning the annealing conditions enhanced the sensitivity to dopamine). Adapted with permission from ref. 62. Copyright 2021 American Chemical Society. [D] Defect engineering to enhance surface reactions (example: charge transfer kinetics are enhanced by introducing defects). Adapted with permission from ref. 63. Copyright 2020 Elsevier. [E] Porosity engineering (example: compared to planar GR, porous GR had 4.2 times higher response to CO_2_ and 10.4 times higher response to NH_3_). Adapted with permission from ref. 64. Copyright 2020 Elsevier.

GR can be functionalized through various methods to enhance its response to bioanalytes (Fig. 3).^13,56–60^ Some of the strategies include doping, defect engineering, surface modification, porosity engineering, and annealing, which are summarized below.

#### Doping

Doping of GR can be achieved using substitutional doping, electronic doping, photodoping, *etc*.^65^ Substitutional doping is often permanent and refers to the intentional introduction of foreign atoms or molecules into the GR structure to modify its electrical, optical, and chemical properties (Fig. 3A).^66^ Doping of GR can enhance its sensitivity and selectivity towards specific biomarkers in biosensing applications. For example, GR was doped with nitrogen to enhance its electrocatalytic activity for hydrogen peroxide reduction, which was then utilized to detect glucose.^61^ In other work, sulphur-doped graphene formed by a solid-state reaction showed high electrocatalytic activity towards the redox reaction of dopamine leading to a low detection limit of 15 nM.^67^ For a thorough review of GR doping, we refer readers to a focused review article by Lee *et al.*^65^

#### Surface modification

Surface modification of GR-based devices usually involves attaching biorecognition elements such as enzymes, antibodies, or nucleic acids or metal nanoparticles and quantum dots onto the GR surface (Fig. 3B).^68^ This is achieved through chemical or physical adsorption, covalent bonding, or layer-by-layer assembly.^69^ Functionalized GR can then specifically interact with target analytes. For example, using the self-assembly of peptides on the graphene surface, an impedance-based sensor for the detection of bacteria on tooth enamel was developed.^70^ For a thorough review of various methods to functionalize GR, we refer readers to focused review articles by Kuila *et al.* and Yang *et al.*^13,59^

#### Annealing

Annealing – a process of heating and cooling the GR material – can also be used as a functionalization strategy (Fig. 3C).^71^ Annealing enables the removal of impurities and defects from GR, thereby improving its electrical and structural properties. Moreover, annealing can induce structural rearrangements in GR, leading to increased sensitivity and selectivity towards biomolecules.^72^ In one work, Butler *et al.* showed that post-deposition annealing of screen-printed GR ink at 300 °C for 30 min under a hydrogen and argon environment led to a dopamine sensor that could detect dopamine down to 5 pM. This was due to the increased hydroxyl groups on the surface, which positively improved the adsorption of dopamine on the GR surface.^62^

#### Defect engineering

Defect engineering involves intentionally creating defects in the GR structure to enhance its sensitivity towards biomolecules (Fig. 3D).^73^ These defects can be introduced through methods such as ion irradiation, chemical functionalization, thermal treatments, or plasma treatments.^74^ The defects not only create additional active sites for biomolecule binding but also alter the electronic properties of GR, leading to improved sensing performance. Defect density estimation of GR materials using Raman spectroscopy^75^ has been used to correlate the dopamine sensing response of GR to the defect density. It was observed that the density of point defects directly improved the sensitivity whereas the grain area corresponding to the line defects increased the baseline.^76,77^ For a thorough review of defects in GR, we refer readers to a review article by Liu *et al.*^74^

#### Porosity engineering

Porosity engineering involves creating pores or channels in the GR structure to increase its surface area and enhance biomolecule adsorption (Fig. 3E).^78^ This can be achieved through techniques such as CVD, electrochemical etching, or physical methods including laser- or plasma-based etching.^79^ In one work, nanoporous graphene was fabricated using microwave plasma CVD and subsequently fragmented using ultrasonication and deposited on a substrate.^64^ Compared to planar GR, porous GR had a 4.2 times higher response to CO_2_ and 10.4 times higher response to NH_3_, highlighting the advantage of nanopores in sensor sensitivity.^80^

### Graphene derivatives

Apart from graphene itself, different derivatives have been synthesized and incorporated into bioanalytical devices. This section provides a brief overview of important GR derivatives.

#### GO and rGO

**Fig. 4:**
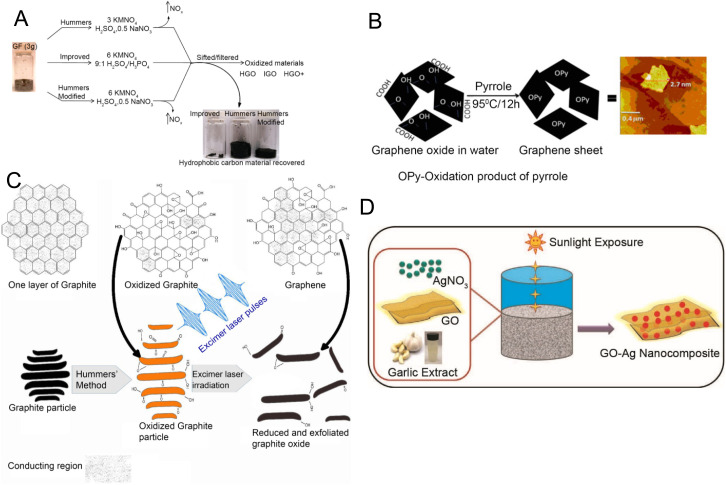
Synthesis methods for examples of graphene derivatives used in electroanalytical devices. [A] Schematic illustration of graphene oxide (GO) synthesis by chemical oxidation. Reprinted with permission from ref. 96. Copyright 2010 American Chemical Society. [B] Synthesis of reduced graphene oxide (rGO) *via* pyrrole process. Reprinted with permission from ref. 97. Copyright 2011 Elsevier. [C] rGO synthesis *via* excimer laser processing. Reprinted with permission from ref. 98. Copyright 2018 Elsevier. [D] Synthesis of GO–Ag (silver) nanocomposite using garlic extract and sunlight. Reprinted with permission from ref. 99. Copyright 2015 Elsevier.

One of the challenges with GR is its insolubility in many solvents.^80,81^ Graphene oxide (GO) and reduced graphene oxide (rGO) provide effective solutions to this challenge.^82–89^ The abundance of oxygen-functional groups in GO makes it soluble in a wide array of solvents.^90^ The synthesis of GO can be achieved by chemical exfoliation of graphite or by oxidation of GR. Primary production methods like the Brodie method^91,92^ and the Staudenmaier method^93,94^ have been phased out due to safety and environmental concerns. Hummers’ method^95^ and its subsequent modifications^96^ (Fig. 4A) are the most prevalent techniques for GO production due to the reduction in toxic gas emissions and improvement in the production yield.

rGO is produced by reducing GO. It has a lower oxidation state and reduced surface reactivity in comparison with its precursor material, which makes it suitable for biomedical applications due to minimal cellular damage.^100^ Factors such as production efficiency, biocompatibility, and low toxicity should be considered when synthesizing rGO that is suitable for biomedical applications.^101^ Hybrid methods that combine photothermal and photochemical treatments using ultraviolet light^102^ or laser techniques^103,104^ introduce a time-efficient and cleaner production process. Another potential way to obtain relatively clean and efficient rGO is the electrochemical reduction method in which rGO can be precipitated from a graphite electrode through changes in voltage.^105^ Thermal reduction relies on heat combined with organic solvents like dimethyl sulfoxide,^106^ dimethylformamide,^107^ or *N*-methyl-2-pyrrolidone^108^ offering an alternative way to reduce GO to rGO. Another approach is the chemical reduction of GO to rGO using reductants such as hydrazine monohydrate,^109^ hydrazine hydrate,^110^ sodium borohydride in high excess,^111^ pyrrole^97^ (Fig. 4B), and others. Besides, green alternatives such as *Delphinium* root extract have been explored for their potential in biocompatible applications.^112^ Photothermal and photochemical treatments using an excimer laser are also developed as faster and relatively cleaner processes (Fig. 4C) to create rGO.^98^

#### Graphene and its derivatives hybridized with other nanomaterials

Another approach to overcome inherent limitations of GR, such as zero band gap, is to focus on hybrid materials by incorporating metal (oxide) nanoparticles, carbon nanotubes (CNTs), transition metal chalcogenides, *etc*. Graphene composites loaded with metal (oxide) nanoparticles have been shown to offer promising improvements in biosensors.^113,114^ Additionally, eco-friendly methods can be used for synthesizing these composites. A study demonstrated the use of natural garlic extract and sunlight to synthesize GO–silver composite, highlighting environmentally sustainable approaches (Fig. 4D).^99^ Other nanomaterial–graphene hybrids have also been developed, such as GR–CNT composites produced using CVD and layer by layer (LBL) assembly. CVD offers precise control over the deposition process, making it an effective method for the preparation of advanced carbon-based nanoelectronics for a wide range of applications.^115–121^ Besides the simplicity and versatility, LBL assembly provides a high degree of control over the structure and composition of the GR–CNT composites.^122–124^ In another work, amine-modified MoS_2_ nanoparticles were encapsulated within GO, which showed remarkable sensitivity and amplified electrochemical signals for the detection of hydrogen peroxide (H_2_O_2_).^125–128^ In another study, a WS_2_–graphene composite for DNA biosensing was synthesised using a hydrothermal approach. The improved sensing performance was associated with enhanced charge transport and a large surface area.^128–132^ These collective developments underscore the immense potential of GR loaded with metal (oxide) nanoparticles and GR-based hybrid structures in biosensor technology.

## Device manufacturing

**Fig. 5:**
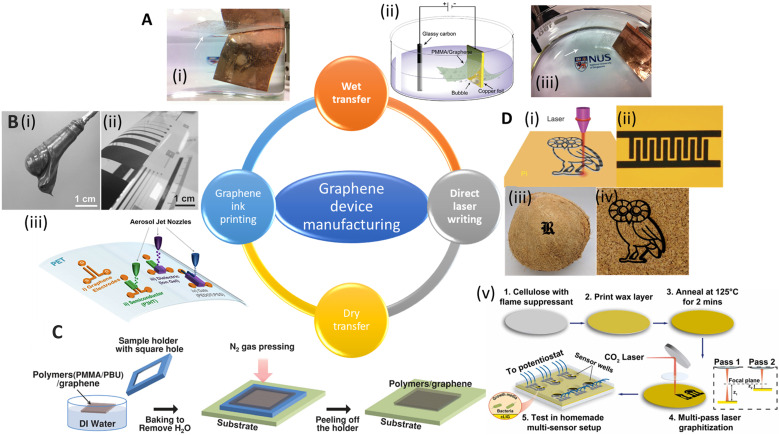
Schematic showing various manufacturing methods for graphene devices. [A] (i) Images of electrochemical exfoliation of graphene from Cu foil. (ii) Schematic diagram of an electrochemical cell used for electrochemical exfoliation. (iii) Another side view of (i). Reprinted with permission from ref. 133. Copyright 2011 American Chemical Society. [B] (i) Image of graphene paste. (ii) Bent PET foil with printed graphene film. Reprinted with permission from ref. 134. Copyright 2015 Wiley-VCH GmbH. (iii) Schematic and optical image of aerosol-jet printed devices using graphene ink. Reprinted with permission from ref. 140. Copyright 2017 American Chemical Society. [C] Illustration of the dry transfer method for CVD-grown graphene. Reprinted with permission from ref. 135. Copyright 2014 Wiley-VCH GmbH. [D] (i) Direct laser scribing to produce laser induced graphene (LIG) on different substrates, such as (ii) polyimide (example: an image of the as-printed LIG interdigitated electrodes). Reprinted with permission from ref. 136. Copyright 2014 Springer Nature. (iii) Coconut shell and (iv) cork. Reprinted with permission from ref. 137. Copyright 2018 American Chemical Society. (v) A schematic overview of the fabrication process for sensors based on cLIG (cellulose-based laser induced graphene). The sensors were used for real time monitoring of the viability and phenazine production by *P. aeruginosa* cells. Reprinted with permission from ref. 138. Copyright 2023 Elsevier.

The preparation of GR-based biosensors has been the subject of various research endeavors, especially for healthcare applications. Typical manufacturing methods include graphene transfer (Fig. 5A), printing (*e.g.*, inkjet printing and screen printing, Fig. 5B), photo/electron lithography (Fig. 5C), and direct writing methods (Fig. 5D).

### Lithography of transferred graphene films

To fabricate GR devices, especially GR-based field-effect transistors (GFETs), GR (usually synthesized using the CVD method) is first transferred from the original substrate to the target substrate. Then lithography is used to transfer patterns of a mask onto graphene by means of etching.^139^ The graphene transfer is done either using wet or dry processes, which are summarized below. The target substrate is usually a silicon wafer for the nanopreparation process. Recently, GR has also been successfully transferred onto flexible substrates to create flexible/wearable devices. Some common flexible substrates used as supports for GR include polydimethylsiloxane (PDMS),^140,141^ polymethylmethacrylate (PMMA),^142^ and polyethylene terephthalate (PET).^143^ For a focused review of transfer processes, we refer the readers to a review article by Ullah *et al.*^144^

#### Wet transfer

The wet transfer method was first developed to transfer graphene grown on metal substrates. During the wet transfer process, the graphene sample is placed in ionic etchants (*e.g.*, ferric chloride, ammonium persulfate) to remove the metal catalyst. In a work by Ameri *et al.*, they used the “wet transfer, dry patterning” process to pattern CVD graphene as a skin tattoo sensor.^145^ Graphene on Cu foil was first spin-coated with PMMA and then placed in the etchant to remove copper; then graphene on PMMA was patterned by a mechanical cutter plotter and peeled off. Wood *et al.* compared different supporting layers including PMMA, poly(phthalaldehyde), poly(lactic acid), and poly(bisphenol A carbonate) (PC).^146^ They found that PC provided the best GR properties without an annealing process. By using chloroform, PC scaffolds can be totally removed at room temperature. Raman spectroscopy and atomic force microscopy studies showed fewer defects and a smooth surface for the PC-transferred graphene.

Traditional wet transfer usually etches the metal layer during transfer. However, this approach produces lots of waste, takes a long time, is challenging to reproduce, and is usually costly for mass production of GR. The electrochemical bubbling transfer method was developed to reuse the metal substrate. In this approach, graphene/Cu was employed as the electrode for water electrolysis (Fig. 5A). The produced gas (O_2_ and H_2_) bubbles generate a peeling force between graphene and the metal.^133^ The electrochemical bubbling transfer method provides a fast, economical way compared to the traditional etching method, which makes this method a better fit for the large-scale production of GR.

While the wet transfer process is the most common method used in laboratories to obtain GR, it usually suffers from contamination by the etchant(s) and/or the supporting polymer, or the metal residues on GR may degrade the electron mobility and may lead to unnecessary doping of GR.^147^ In addition, the etching chemicals are not environmentally friendly. To address these challenges, dry transfer techniques have been developed as summarized below.

#### Dry transfer

To overcome the issues associated with the wet transfer process, dry transfer was developed as an alternative method to transfer high-quality GR. Thermal release tape was first used to dry transfer epitaxial graphene to SiO_2_ substrate in 2010.^148^ Kim *et al.* developed a dry transfer method using a polymeric bilayer of PMMA and polybutadiene (PBU), where PBU and PMMA were spin-coated on CVD-grown graphene in sequence (Fig. 5C).^135^ The PBU layer reduces charged impurity scattering from PMMA and changes the Fermi level. They also fabricated a flexible GFET on the polyimide substrate using this dry transfer method operating at a low supply voltage of 4 V. However, cracks are usually found in GR during the delamination step, and a universal route and transfer material are needed for commercialization.

**Fig. 6:**
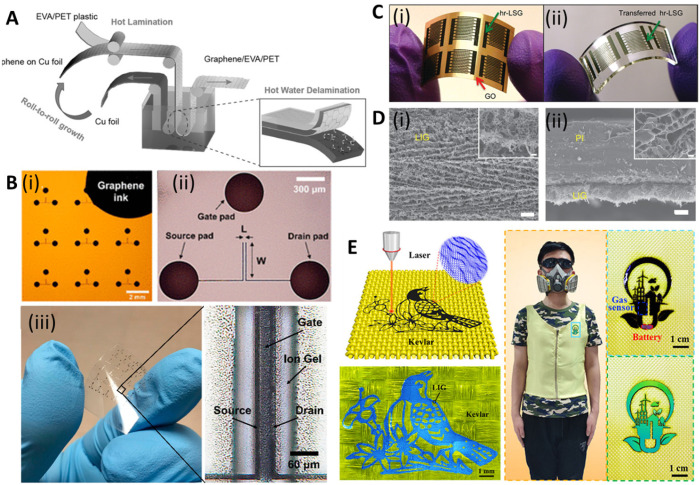
Scalable and printable graphene-based electroanalytical devices. [A] Illustration of roll-to-roll delamination of copper and graphene onto EVA/PET substrate. Reprinted with permission from ref. 149. Copyright 2015 Wiley-VCH GmbH. [B] (i) Image of an electrolyte-gated transistor (EGT), selectively printed with graphene ink, with *L* = 20 and *W* = 400 μm. (ii) Microscope image of a single device in (i). (iii) Photograph of flexible EGTs printed on PET and optical image showing printed layers of source, drain, ion gel, and gate. Reprinted with permission from ref. 150. Copyright 2017 American Chemical Society. [C] (i) Image of interdigitated electrodes fabricated using highly reduced laser scribed graphene (hr-LSG) (ii) Image of hr-LSG after transfer to PDMS. Reprinted with permission from ref. 151. Copyright 2012 American Chemical Society. [D] SEM images of (i) LIG film and (ii) its cross-section. Scale bar: 10 μm and 1 μm in the inset. Reprinted with permission from ref. 136. Copyright 2014 Springer Nature. [E] (i) Schematic of the preparation of graphene/Kevlar textile. (ii) Intelligent clothing integrated with the NO_2_ gas sensor based on the graphene/Kevlar. Reprinted with permission from ref. 152. Copyright 2020 American Chemical Society.

For large-scale and fast transfer to meet market demands, a roll-to-roll manufacturing method was developed. Bae *et al.* reported a way to transfer large-scale CVD-grown graphene using the roll-to-roll method in 2010.^143^ In this process, the graphene films are detached from the tapes and released to the substrates by thermal treatment. The transfer rate was 150–200 mm min^−1^ and a wet-chemical doping process could also be added to the process. In 2015, Liu's group reported a modified roll-to-roll clean transfer of CVD-grown graphene from copper to the ethylene vinyl acetate/poly(ethylene terephthalate) (EVA/PET) plastic substrate (Fig. 6A).^149^ This process was achieved by water penetrating between graphene and the copper oxide layer. They also used the transferred graphene to manufacture a wearable transparent and flexible triboelectric nanogenerator to show good conductivity and high transparency of the transferred graphene. The roll-to-roll method is compatible with the current industrial set-up for low-cost and large-scale transfer, but it can only transfer to flexible substrates, preventing its extensive application.

### Printing techniques

Although lithography with transferred GR films has shown its reproducibility and processability in manufacturing GR-based devices, there are some disadvantages that hinder their practical applications, *e.g.*, they need complex and expensive tools and highly trained personnel. In addition, lithography mask residues often result in contamination of the devices and the high-energy etching processes may introduce defects into GR.^139^ In contrast, printing techniques offer a suite of desirable properties for printed electronics, including easier preparation, high electrical conductivity, flexibility, and robust mechanical, chemical, and environmental stability.^153,154^

#### Printing graphene ink

To explore an environmentally friendly, affordable, and scalable GR synthesis method that has the potential to fabricate electrical devices on a large scale, many printing methods are developed, including screen-printing, spin-coating, and inkjet and aerosol-jet printing. Initially, exfoliated GR in liquid media was directly used for printing the devices. Much effort has been put into the exfoliation method and the solvent to get high-quality GR.^155–157^ Secor *et al.* developed GR ink using ethanol as the solvent and ethyl cellulose as the stabilizer.^158^ After annealing at 250 °C for 30 min, the ink was used to print the GR lines on the hexamethyldisilane-treated Si/SiO_2_ substrate, yielding the printed GR with a resistance of 260 Ω sq^−1^. In 2015, Arapov *et al.* reported GR/polymer dispersions with 30 Ω sq^−1^ resistance at 25 μm thickness *via* screen printing (Fig. 5B(i) and (ii)).^134^ The printed pattern only needed to dry at 100 °C for 5 min. Although significant progress has been made in the commercial application of GR inks, some bottlenecks still limit their widespread use. For example, the GR mass content in ink is still low compared with other conductive inks because of the low dispersibility of GR in many non-toxic solvents.^159^ In addition, GR inks usually need to be filtered to remove the large flakes that may block the nozzles of inkjet printing. The low concentration and small size increase the resistivity of the printed devices. To overcome these issues, Jabari *et al.* reported a GR/Ag hybrid ink to aerosol-jet print conductive patterns.^160^ The resistance was lower than 1 Ω sq^−1^, about 100 times lower that of the GR pattern and 3 times lower than the Ag nanoparticle ink alone.

Despite their widespread use, the resolution of traditional inkjet printers is poor, about 30–50 μm, limiting their application in printing high-resolution electronic devices (*e.g.*, flexible transistors). Song *et al.* demonstrated a method to fabricate high-resolution patterns using the transfer printing of GR ink with silicon molds^150^ (Fig. 5C(iii)). The smallest line width and spacing of the patterns were 3.2 μm and 2.7 μm, respectively. They further transferred the pattern onto PET and fabricated flexible electrolyte-gated transistors to show their great mechanical robustness and electrical properties (Fig. 6B). GR ink can also be spin-coated on flexible substrates. Muralidharan *et al.* reported a flexible electrochemical dopamine sensor by spin-coating GR ink onto polyimide followed by an annealing process.^161^ The sensor showed a limit of detection (LOD) of 100 nM of dopamine in phosphate buffered saline (PBS) and a linear range of up to 1 mM. Further study showed that the LOD was improved to 5 nM when the sensor was treated with copper sulfate solution to dope GR. Interfacing the sensor with a wireless system with an on-chip integrated potentiostat also made the sensors suitable for point of care monitoring. For a more focused review of printable GR devices, we refer readers to Htwe *et al.* and Fisher *et al.* Htwe *et al.* summarized recent progress on using GR ink in flexible electronics applications and compared different flexible substrates used in wearable devices.^162^ In addition, Fisher *et al.* provided a survey of state-of-the-art GR aerosol-jet printing for sensing applications.^163^

#### Direct laser writing

Emerging in 2012, the direct laser writing (DLW) method enabled micro-/nanometer patterning of GR from GO film.^151,164^ GO shows good solubility in water and can be easily processed to form free-standing films. The energy from the laser reduces GO to rGO, dubbed as laser-scribed graphene or laser-induced graphene (LIG), which benefits from the high charge mobility and large surface area; it was used for energy storage (*e.g.*, supercapacitor and lithium-ion batteries) and flexible electrodes (Fig. 6C).

In 2014, Tour group reported a method that used CO_2_ infrared laser DLW to convert various flexible substrates, *e.g.*, polyimide (PI), into porous 3D LIG films (Fig. 5D(i) and (ii)).^136^ The gas produced by the elevated temperature due to the laser source forms porous structures with more readily accessible surfaces (Fig. 6D). It is challenging to achieve precise high-resolution thermal treatments with conventional thermal treatments, but they can be easily accomplished with a focused laser beam by inducing photothermal or photochemical reactions with the substrate. These localized reactions strongly depend on the laser power, processing parameters, and material properties. Multiple lasing can create the LIG pattern on naturally occurring substrates such as cloth, paper, potato skins, coconut shells, and cork (Fig. 5D(iii) and (iv)).^137^ DLW is a maskless, eco-friendly, and cost-effective method compared to conventional GR functionalization methods.^165^ It also overcomes the delamination issues that usually cause failures in traditional flexible GR devices made *via* the transfer method.

Recently, LIG-based devices have become attractive alternative materials in the field of electrochemical biosensing. For example, Butler *et al.* developed a paper-based electrochemical sensor using cellulose-based laser-induced graphene using a multi-setup laser writing process (Fig. 5D(v)). The 3-D structure of paper closely replicates the native cellular environment, which is favourable for the growth of cells.^138^ In other work, Gao's group developed a wireless COVID-19 immunoassay called RapidPlex using LIG.^166^ Cheng's group successfully developed a series of LIG-based sensors for multiple applications including on-body glucose sensing,^167^ temperature and motion detection,^168^ NO_2_ detection,^169^ and self-powered sensing.^170^ Wang *et al.* implemented the LIG writing process on Kevlar textile in air and designed smart protective clothing based on the graphene/Kevlar NO_2_ sensor (Fig. 6E).^152^ Directly written LIG on cloth enables facile preparation of smart textile electronics.

It should be noted that various laser sources have been used to prepare carbon-based materials. While polyimide is optically transparent at the CO_2_ laser wavelength that is commonly used in developing LIG-based devices (10.6 μm), it is not transparent in the UV region. Thus, to create devices with a smaller feature size, lasers in the UV range are used. For example, Carvalho *et al.* demonstrated writing LIG using a UV pulsed laser (355 nm) to develop pulse wave sensors.^171^ Morosawa *et al.* showed laser graphitization of cellulose nanofiber using a high-repetition femtosecond laser with a central wavelength of 522 nm, achieving a conductivity of 6.9 S cm^−1^, which was about 100 times higher than that previously reported.^172^

## Transduction mechanisms of GR-based electroanalytical devices

Sensor transduction mechanisms are pivotal in their functionality, as they convert the material-analyte interaction into a measurable signal.^173^ In the context of GR-based electroanalytical sensors, the main transduction mechanisms include electrochemical impedance spectroscopy (EIS), field-effect transistors (FETs), various voltammetry techniques, electrical conductivity-based sensors, and multimodal methods.^174^

The EIS method measures the impedance of a system across a range of frequencies, reflecting changes in the electrical properties of the system (interface and bulk) upon analyte interaction. Methods based on FET utilize changes in the electric field to modulate conductivity within a transistor channel in the presence of an analyte. Voltammetry techniques (steady state and pulsed methods) involve potential sweeps and measuring the subsequent current to deduce the concentration of an analyte. Electrical conductivity-based sensors detect alterations in conductivity when an analyte interacts with the sensor's surface. Lastly, multimodal methods such as photoelectrochemical and Raman–electrochemical signals combine several techniques to improve the analytical accuracy.^174^ In the following, a brief overview of each method is provided.

### Electrochemical impedance spectroscopy (EIS)

The EIS technique investigates the behaviour of electrochemical systems by imposing a sinusoidal voltage or current and observing the system's response across a spectrum of frequencies. EIS measures the complex impedance of a system (*Z*), which includes both faradaic and non-faradaic components. The impedance is depicted graphically using Nyquist or Bode plots (*i.e.* imaginary *vs.* real components or impedance magnitude and phase against frequency, respectively).^175^ One of the hallmarks of EIS is its capacity to correlate the measured total impedance to equivalent circuit models comprised of multiple impedance components, such as interfacial charge transfer resistance due to faradaic processes (*R*_ct_), double layer capacitance (*C*_dl_), and Warburg impedance (*Z*_w_, which is associated with mass transport/diffusion) – each providing important insights into the system under study. By modelling and fitting EIS data to these circuit components, a multitude of biosensors have been developed for a variety of applications including *in vitro* detection of analytes,^176–178^ activation of ionic pumps in bacterial cells,^179^ and mechanisms involved in the interaction of cells with environmental triggers such as cancer drugs,^180–183^ antibiotics,^184,185^ and heat shock,^186^ among others. A thorough review of EIS-based biosensors can be found elsewhere.^187–189^

**Fig. 7:**
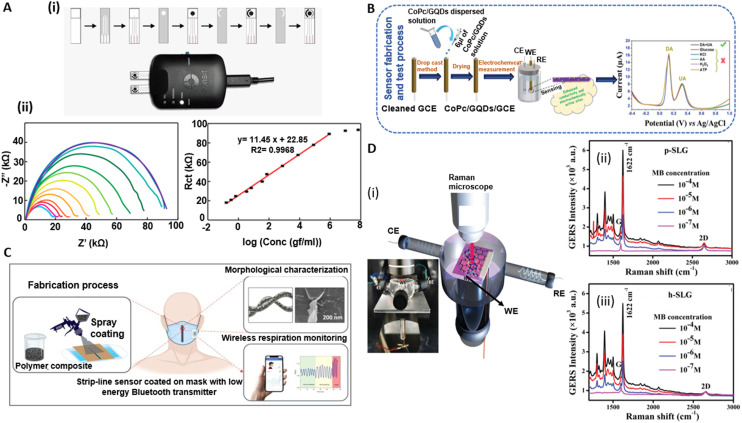
Electroanalytical devices based on electrochemical impedance spectroscopy (EIS), voltammetry, electrical conductivity, and multimodal methods. [A] (i) Preparation process to create graphene-based electrochemical test strips for SARS-CoV-2 detection. (ii) EIS data (Nyquist curves) and the extracted charge transfer resistance, *R*_ct_, for different analyte concentrations. Reprinted with permission from ref. 34. Copyright 2023 MDPI Diagnostics. [B] Performance of CoPc/GQDs/GCE electrodes for simultaneous dopamine (DA) and uric acid (UA) detection, showing low detection limits and high selectivity. Reprinted with permission from ref. 198. Copyright 2022 Wiley Advanced Materials Interfaces. [C] Graphene nanoplatelets (GR-NPs) utilized for enhancing the electrical and piezoresistive properties of surgical mask sensors, with an emphasis on the inverse relationship of GR-NP content to gauge factor. Reprinted with permission from ref. 199. Copyright 2023 Elsevier Materials and Design. [D] (i)–(iii) Application of pristine and hydrogenated single-layer graphene in graphene-enhanced Raman spectroscopy (GERS), demonstrating the modulation of GERS signals and sensitivity through doping with holes. Reprinted with permission from ref. 200. Copyright 2022 Wiley Advanced Materials Interfaces.

GR's high electrical conductivity makes it a promising material for the development of EIS-based devices. Different electrode materials based on GR have been investigated for developing EIS-based sensors, including GR nanoplatelets,^190^ LIG,^191^ and GR ink.^192^ Moreira *et al.* demonstrated an aptasensor based on EIS.^193^ The proposed aptasensor for the detection of SARS-CoV-2 in human saliva was fabricated using LIG. A comprehensive analysis of EIS signals identified the optimal response along with suitable cutoff frequencies. Their findings highlighted the significance of variables such as the negative phase (−*ϕ*), total impedance (*Z*), real and imaginary impedance (*Z*′ and *Z*′′), as well as the real and imaginary capacitance (*C*′ and *C*′′) in establishing the GR-aptasensor baseline and selecting cutoff frequencies. In other work, Akbari *et al.* developed EIS-based sensors for the detection of microRNA (miR-223), which are one group of pertinent molecular biomarkers for the early detection of cancers. The sensor electrode was comprised of a layer of gold nanoparticles deposited on GO on fluorine doped tin oxide (FTO)-coated glass substrates. The sensors achieved an LOD of 0.012 aM. The sensor was utilized in practical applications for miR-223 detection in human serum and demonstrated an extraordinary capacity for accurate detection, as evidenced by a minimal percentage of relative standard deviation (RSD = 5.7%). The linear detection range of miR-223 using this sensor was from zM to nM. Additionally, the biosensor's selectivity was thoroughly evaluated using alternative miRNAs, particularly miR-486, as mismatch targets to discern the selectivity performance.^194^ In another work, Anghel *et al.*^195^ utilized vertically oriented graphene (VGR)-based electrochemical sensors to distinguish between two types of human colon adenocarcinoma cells, SW403 and HT29, with high and low invasiveness. The sensors were tested on cell concentrations ranging from 10^4^ to 10^6^ cells per mL. Through EIS, they discovered significant differences in electrical properties between the cell types. HT29 cells demonstrated lower electrical charge transfer resistance and higher permittivity and conductivity, attributed to their highly folded membrane surface. The study particularly noted that SW403 cells, at a concentration of 104 cells per mL, showed the highest charge transfer resistance (*R*_ct_ = 3250 Ω) and a lower capacitance (*C*_dl_ = 6.38 μF) compared to higher concentrations of the same cells and HT29 cells. This observation was linked to SW403 cells forming large conglomerates, impeding electrical current flow. The EIS analysis effectively revealed how tumor cells captured at the electrode–molecule interface obstructed electron transfer, enabling impedance-based measurement of tumor cells. In another work, Tukimin *et al.* developed an electrochemical sensor using poly(3,4-ethylenedioxythiophene)/rGO (PrGO) demonstrating superior sensitivity and selectivity for uric acid (UA) detection in the presence of ascorbic acid (AA).^196^ Ehsan *et al.*^197^ developed a graphene-based EIS sensor for the detection of SARS-CoV-2. The sensor utilized IgG anti-SARS-CoV-2 spike antibodies to quantify viral antigens in various media. They used high conductivity GR/carbon ink, which reduced background impedance, thereby extending the dynamic detection range. Key performance metrics for this sensor included an impressively low limit of quantification of 0.25 fg mL^−1^. The sensor's linear detection range was significantly enhanced through biological entity-based antibody immobilization, spanning from 0.25 fg mL^−1^ to 1 ng mL^−1^. This range is particularly noteworthy as it marks a substantial improvement over conventional methods such as ELISA platforms. The sensor's selectivity was rigorously tested against H1N1 flu antigens, where it showed no significant response, indicating a high specificity for COVID-19 antigens (Fig. 7A(i) and (ii)).

### Voltammetry techniques

Voltammetry methods include cyclic voltammetry (CV, where the potential between electrodes is cyclically varied to study redox reaction kinetics), linear sweep voltammetry (LSV, involving a linear potential sweep to analyze electron transfer reactions), and pulse-based methods such as differential pulse voltammetry (DPV, which uses periodic pulses superimposed on a linear potential sweep for enhanced resolution of closely spaced redox species), among others. In these techniques, a three-electrode system is usually used: a working electrode (WE, often modified with GR-based material for increased sensitivity and stability), a reference electrode (RE, typically Ag/AgCl ink in on-chip systems), and a counter electrode (CE, commonly platinum or gold).^175^ Among various steady state voltammetry methods, CV is used extensively to decipher the underlying electrochemical mechanisms, electron transfer kinetics, and diffusion processes, allowing researchers to elucidate the properties of sensing materials. On the other hand, pulsed voltammetry, like DPV, and square wave voltammetry (SWV) offer enhanced sensitivity and analytical capabilities. These methods achieve improved performance by applying pulses that decrease background noise and increase signal clarity, allowing for the detection of trace-level substances. These techniques also provide faster, high-resolution analysis, making them suitable for complex mixtures and a wide range of applications, from environmental to clinical diagnostics.^201^ A thorough overview of voltammetry-based biosensors can be found in review articles by Yuwen *et al.*, Majer-Baranyi *et al.*, and Bano *et al.*^202–204^

Several voltammetry-based sensors utilizing GR and its derivatives/hybrids have been reported. For example, Saisahas *et al.* developed electrochemical paper-based analytical devices (ePAD) enriched with graphene ink and modified with polyaniline (PANI) for the detection of xylazine, a veterinary sedative, using DPV. Enhanced with graphene and polyaniline, the ePAD ensures efficient charge transfer with a larger surface area. It showcases a low detection limit of 0.06 μg mL^−1^ and a reliable performance with less than 5% deviation. Practical testing confirmed its accuracy, with 84–105% recovery in beverage samples.^205^

Another study used GR quantum dots (GQDs) coupled with cobalt phthalocyanine (CoPc) to modify glassy carbon electrodes (GCEs).^198^ The resulting CoPc/GQDs/GCEs showed superior electrocatalytic activity for the simultaneous detection of dopamine (DA) and uric acid (UA) in PBS, outperforming traditional CoPc electrodes. The CoPc/GQDs/GCE showed reversible DA redox peaks and an irreversible UA oxidation peak on CV curves displaying heightened sensitivity and selectivity for simultaneous DA and UA detection. Notably, the sensor achieved low detection limits of 21 nM for DA and 145 nM for UA, operating within distinct linear ranges: 2.91–33.38 μM and 45.39–164.2 μM for DA (with an *R*^2^ = 0.9809 and 0.9922, respectively) and 10.76–3003 μM for UA (*R*^2^ = 0.9901). Additionally, selectivity tests showed minimal interference from common substances, maintaining a relative error within ±5.5%, confirming its analytical precision. Furthermore, stability testing revealed impressive durability, with response retention of 87.9% for DA and 92.3% for UA over 13 days, underscoring the sensor's potential for long-term biomedical and clinical usage (Fig. 7B).

In another work, a LIG-based sensor for the detection of 4-nitrophenol (4-NP, a water pollutant) was developed. 4-NP was analyzed in a 0.1 M PBS solution using the LSV method. The sensors exhibited linear behavior in two concentration ranges (0.15 to 1 μM and 2.5 to 100 μM) with a LOD of 95 nM. The sensor displayed good selectivity for 4-NP, even in the presence of isomers and other phenolic compounds and in sewage samples with different 4-NP concentrations.^206^

### Electrical conductivity

Electrical conductivity represents a material's capacity to conduct electric current. Upon occurrence of the event of interest (such as humidity change, biomolecule capture, or change of strain), the conductance (inverse of resistance) changes. For example, Wu *et al.*^207^ utilized crumpled GR, derived from heat-induced transformation of polystyrene-coated CVD-grown GR, for the sensitive detection of the cancer biomarker miRNA-21. This unique structure, enhanced through low-damage plasma treatment, increases the surface area and creates an optimized microenvironment for target capture, notably improving the sensor's sensitivity and selectivity. The biosensor achieved an impressive LOD of 1.74 pM and a high degree of linearity from 10 nM to 1 pM. Practical application assessments conducted in complex biological media, specifically undiluted human serum albumin (HSA) and PBS, confirmed the sensor's robust performance and low interference, maintaining effective biomarker detection under real-world conditions. Stability tests further revealed that the sensor's performance remained above 95% over a one-week period, indicating its suitability for sustained use in clinical settings.

In another work, graphene nanoplatelets (GR-NPs) were used to enhance the capabilities of surgical mask sensors, specifically focusing on electrical conductivity and piezoresistive behavior.^199^ GR-NPs, due to their excellent electrical properties, were used to create conductive pathways on the mask's surface. Three types of masks, each with varying GR-NP concentrations, were subjected to electromechanical tests to examine changes in resistance under structural deformation. The increase in resistance under applied strain, particularly evident in the low GR-NP sample, was due to two primary mechanisms: (i) the disconnection mechanism involved a reduction in overlapped areas between GR flakes under strain, and (ii) the tunneling effect allowed electrons to cross the nonconductive polymer matrix, with tunneling resistance increasing proportionally to the GR-NP aggregation distance. The gauge factor, GR = (Δ*R*/*R*0/*ε*), representing the sensitivity of the sensors, was inversely related to the GR-NP content. The GR-NP-based strip lines demonstrated a fast response time (∼42 ms) and exceptional reproducibility and stability when used as a respiration sensor. Despite changes in temperature and humidity, the sensor's performance remained consistent, thereby validating its potential in healthcare applications (Fig. 7C).

Beniwal *et al.* used graphene–conductive carbon (GR-C) to construct humidity sensors *via* a screen-printing method.^208^ The screen-printing process enabled the creation of sensors with different layer numbers. The layers’ composition significantly influenced the sensors’ electrical conductivity, impacting the sensors’ response to humidity. Researchers observed that sensor resistance increased proportionally to relative humidity (RH) levels, ranging from 35%RH to 91%RH. Each sensor configuration showed distinct baseline resistances and resistance change rates due to their layer differences, with the single-layer sensor exhibiting superior electrical conductivity changes in response to varying humidity levels. The sensor's response and recovery times were the fastest for a one-layer sensor, underscoring its enhanced performance.

### Multimodal methods

Apart from the methods summarized above, there are a variety of multimodal methods based on GR for biosensing. Two of the most studied ones include photoelectrochemical (PEC) and Raman–electrochemical methods. The PEC technique involves the generation of photocurrents when a semiconductor absorbs light, leading to electron–hole pair creation. In contrast, Raman–electrochemical methods provide insights into molecular vibrations in electrochemical environments, capturing changes in the Raman spectra due to alterations in the oxidation state or molecular structure. Recent studies have reported the combination of these techniques using GR-based biosensors. These sensors exploit the unique electronic properties of GR and its enhanced Raman and PEC responsiveness. When combined, they offer sensitive, rapid, and multiplexed detection of biomolecules, holding great promise for non-invasive diagnostics and real-time health monitoring.^209^

In one of the notable contributions, a GR-based PEC immunosensor was developed, featuring green synthesized rGO-Au as the substrate.^200^ This study illuminated the sensor's promising capability to detect the S100β biomarker, an indicator of neurological disorders. rGO-Au was employed to create a biocompatible microenvironment for the immobilized antibodies, maintaining their activity and significantly enhancing the sensor's long-term stability. The immunosensor exhibited a “signal-on” response trend when subjected to different S100β concentrations. This trend denoted that the photocurrent intensity rose proportionately with increased S100β biomarker concentration during incubation with labelled anti-S100β. The immunosensor demonstrated a robust linear response over a wide dynamic linear range (DLR) of 0.25 to 10 000 pg mL^−1^ S100β, with an impressively low LOD of 0.15 pg mL^−1^. Furthermore, this LOD surpassed the performance of prior GR-based immunosensors, and the DLR was higher than that of a poly(ethyleneimine)-based sensor; this was attributed to the effectiveness of the rGO-Au platform. Notably, the sensor was resilient to interference from common antigens such as HSA and human immunoglobulin G (HIgG), emphasizing its selectivity in S100β detection.

One of GR's distinctive applications is in GR-enhanced Raman spectroscopy (GERS), which is rooted in a charge transfer mechanism that fosters interaction between adsorbed molecules and the GR substrate. Contrary to traditional surface-enhanced Raman spectroscopy (SERS), GERS offers a more stable and reproducible Raman signal through a chemical mechanism, highlighting its potential over other methodologies. In this context, Kaushik *et al.* recently showcased the use of pristine (p-) (Fig. 7D (ii)) and hydrogenated (h-) (Fig. 7D (iii)) graphene in devising GR-enhanced spectro-electrochemical sensors (GE-SPECSs).^210^ Their study revealed that hole-doped h-graphene exhibited superior GERS signals compared to p-graphene, achieving a LOD of around 10^–7^ M. By capitalizing on the adjustable work function of graphene, they demonstrated the ability to modulate the GERS signal and probe various oxidation states of molecules through the application of appropriate external potentials (Fig. 7D (i)).

### Biosensors based on graphene field effect transistors (GFETs)

Field effect transistors, particularly electrolyte-gated transistors (EGTs) are central to bioelectronic devices. They can transduce and amplify biological signals into electronic ones at low voltages making them suitable candidates for low power biosensing. With three terminals (source: s, drain: d, and gate: g), the current between the source and the drain (*I*_ds_) is modulated by applying a voltage to the gate electrode (*V*_gs_), which directly contacts both the electrolyte and the transistor channel. By applying voltage, the ions drift from the electrolyte toward the channel material, leading to alterations in the electronic charges within the channel influencing its conductivity. This results in modulation of the electronic current flowing through the transistor channel. The unique design of EGTs facilitates their operation at low voltages compared to conventional FETs, making them suitable for cell monitoring,^211^ electrophysiology,^212^ and *in vitro* biosensing,^213^ among others.

The operating characteristics of GFETs are commonly described through three foundational curves: transfer characteristics (*I*_ds_–*V*_gs_), output curves (*I*_ds_–*V*_ds_), and time-series measurements. In GR-based devices, the transfer characteristic – often depicted as a V-shaped graph – is generated by changing the gate voltage, *V*_gs_, while maintaining a constant source–drain voltage, *V*_ds_. This curve is crucial for understanding key parameters, mainly the transconductance (*g*_m_ = (*W*/*L*)*μC*_g_*V*_ds_, where *W* and *L* are the width and length of the GR channel, *μ* is the mobility of charge carriers and *C*_g_ is the gate capacitance). Transconductance itself is influenced by various factors, such as the dimensions of the channel and the gate capacitance. The choice of the gate configuration also impacts the performance of a GFET sensor. For applications involving liquid samples, an electrolyte gate is frequently preferred due to its ability to facilitate efficient electrical measurements directly within the sample medium. On the other hand, back-gate configurations are better suited for sensing volatile compounds in gaseous media.^214^

Most GFET biosensors operate mainly based on the charge transfer principles and the shift of the Dirac point/voltage (*V*_dirac_), which is the *V*_gs_ at which the minimum conductivity point (*σ*_min_) occurs in GR.^215^ In a pristine state, this point occurs at *V*_gs_ = 0; however, it can shift upon the functionalization/doping of GR or its interaction with other molecules.^216,217^ One mechanism that causes shift in the Dirac point involves direct charge transfers between redox probes and targets, resulting in either n-doping or p-doping effects on GR. This alteration affects GR's Fermi level, leading to changes in conductivity and subsequently shifting the Dirac point.^218^ Another factor influencing this shift is the electrostatic gating effect, where probe–target binding induces a local external voltage drop across the GR channel due to the accumulated charges. This effect shifts the Dirac point similarly to the doping effect, contributing a significant role to the sensing mechanism by GFET.^219,220^

**Fig. 8:**
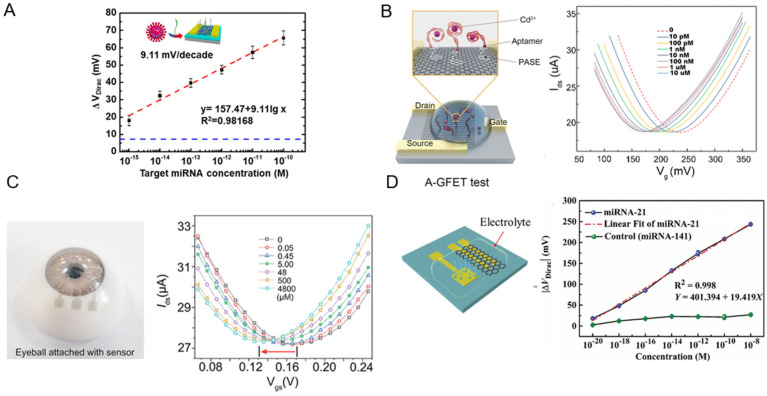
Graphene-based field effect transistors (GFETs) for biosensing. [A] A GFET functionalized with poly-l-lysine (PLL) for detection of miRNA and SARS-CoV-2 RNA. Δ*V*_Dirac_ as a function of analyte concentration is plotted. Reprinted with permission from ref. 221. Copyright 2022 American Chemical Society. [B] A comprehensive three-stage process showing efficient oil–water separation and Cd^2+^ detection *via* an aptamer-functionalized GFET. Reproduced with permission from ref. 222. Copyright 2022 the Royal Society of Chemistry. [C] A wearable nanosensor, utilizing a single-layer GR channel, leverages the Dirac point shift to detect variations in l-cysteine concentrations in artificial tear. Used with permission from ref. 223. Copyright 2022 Wiley. [D] Solution-gated graphene transistors functionalized with ssDNA probes offer a label-free approach for miRNA-21 detection, using Dirac voltage shifts for highly sensitive prostate cancer diagnosis. Used with permission from ref. 224. Copyright 2022 Wiley.

In one example, Gao *et al.* conducted a study where they synthesized monolayer graphene *via* chemical vapor deposition to fabricate poly-l-lysine (PLL)-functionalized graphene field-effect transistor biosensors (Fig. 8A).^221^ These devices exhibited the ability to detect specific miRNA sequences associated with breast cancer (miR-4732, miR-191, miR-21, miR-125) and SARS-CoV-2 virus sequences. The biosensor showed a LOD of 1 fM, demonstrating its potential for physiological applications. The readout method for the system was based on changes to the *V*_dirac_ of the biosensors, which served as a crucial indicator of successful detection. The Dirac voltage shifted up to ∼60 mV to the left when exposed to 100 pM of fully complementary SARS-CoV-2 RNA. This pronounced shift implied a robust interaction between the graphene surface and SARS-CoV-2 RNA, which led to change of the electronic properties of graphene and as a result, shift of the Dirac point. The shift toward the left indicated that the RNA interaction was inducing electron-like (n-type) behaviour in graphene.^221^

Zhang *et al.* used single-crystal GR in an electrolyte-gated GFET biosensor.^225^ The sensing was based on a dual detection mechanism employing ultra-high-frequency (UHF) interferometry and monitoring the variation in graphene resistance. The binding of biotin to streptavidin and the interaction between complementary peptides (E and K) were explored in the two model systems in this study. The experiments were conducted in PBS solution at a neutral pH, with a non-covalent labelling technique being used to attach biotin and peptide E onto the surface of the graphene layer. The LOD for streptavidin was remarkably low at 1 × 10^–9^ M, enhancing its physiological relevancy. To enhance sensitivity, the impedance-matching circuits were integrated into the system setup. The GR surface was functionalized with biotin or peptide E, linked *via* a 4 nm long pyrene–polyethylene glycol linker. This functionalization led to a negative (leftward) shift in the Dirac point by around 100 mV, which indicated a change in carrier concentration. Notably, the transconductance and carrier mobility decreased in the electron-dominant region post-functionalization; however, they remained stable in the hole-dominant region. This implied that functionalization introduced more scattering for electrons than holes. Additionally, the UHF signal showed abrupt changes due to its sensitivity towards GR's electrical properties and local capacitance fluctuations upon molecular interactions.^225^

In another work, Wang *et al.* developed a graphene FET functionalized with aptamers (A-GFET) to detect cadmium ions (Cd^2+^) in oily wastewater (Fig. 8B).^222^ This A-GFET showed a remarkable detection limit of 0.125 picomolar (pM) significantly surpassing the World Health Organization's permissible concentration of Cd^2+^ in drinking water. The readout system was based on changes to the A-GFET's electrical signal upon Cd^2+^ ion interaction. As the concentration of Cd^2+^ increased, the graphene transfer characteristic curve consistently shifted to the negative direction along the *x*-axis of the characteristic curve of A-GFET. Besides, an integrated photoacoustic alarm system alerted when the Cd^2+^ concentration exceeded a predetermined threshold.^222^

Huang *et al.* fabricated an innovative transparent wearable GFET biosensor (Fig. 8C).^223^ They employed a PET substrate with a single-layer GR channel and transparent WO_3_/Au/WO_3_ electrodes as drain, source, and gate in order to detect l-cysteine at mildly acidic to neutral pH. To detect changes in biomarker concertation, a shift in the Dirac point is exploited. As the l-cysteine concentrations increase from 0 to 4800 × 10^–6^ M, the Δ*V*_Dirac_ of the biosensor increases by 22 mV to the left. By accurate tracking of the Dirac point through the optimized procedure, LODs as low as 0.043 × 10^–6^ M in artificial tears and 0.022 × 10^–6^ M in undiluted sweat were achieved.^223^

Xue *et al.* introduced a graphene-based bioelectronic sensing platform employing the Dirac point for detecting multiple ions such as potassium (K^+^), sodium (Na^+^), and calcium ions (Ca^2+^).^226^ The sensor array featured a 30 × 30 μm graphene channel and two titanium/gold (Ti/Au) source/drain electrodes, functionalized with ion-selective membranes (ISMs) for K^+^, Na^+^, and Ca^2+^.^226^ Machine learning algorithms were employed for data analysis, resulting in excellent sensitivity and reversibility. Interestingly, Nernstian slopes of −54.7 ± 2.90 mV per decade for K^+^, −56.8 ± 5.87 mV per decade for Na^+^, and −30.1 ± 1.90 mV per decade for Ca^2+^ were achieved through this approach. Furthermore, with negligible sensitivity drifting over six months, the sensor exhibited excellent reversibility and long-term stability.

Deng *et al.* fabricated a solution-gated graphene transistor (SGGT) biosensor for rapid detection of miRNA-21, which is a significant biomarker for early prostate cancer diagnosis (Fig. 8D).^224^ High-quality CVD graphene was grown as the conductive channel of a SGGT for detecting different concentrations of miRNA-21. As the concentrations of the miRNA-21 target increased, the transfer curve moved toward a positive gate voltage due to the negative charge carried by miRNA in the electrolyte solution. By applying single-stranded DNA (ssDNA) probes immobilized on an Au gate electrode, the biosensor achieved a LOD of 10^–20^ M. Importantly, the device was able to distinguish cancer patients from the control group, surpassing the conventional prostate-specific antigen detection technique commonly used for this purpose.

In summary, FET-based biosensors, particularly GR variants, exhibit high sensitivity and adaptability in detecting a wide range of biomolecules. Despite their challenges such as complex manufacturing processes, ionic screening interference, pre-treatment requirements, and shelf-life, these devices offer promising potential in advancing point-of-care sensing. A thorough overview of GFET-based biosensors can be found in review articles by Krishnan *et al.* and Dai *et al.*^227,228^

## Applications

### *In vitro* assays

GR-based sensors have been developed for *in vitro* detection of various important small biomolecules (such as AA, DA, and UA, which are frequently found together in body fluids^229^), hormones (*e.g.*, cortisol, ghrelin, and peptide YY),^230,231^ proteins (*e.g.*, antibodies, spike protein),^232,233^ and pathogens (bacteria, virus). In the following, we provide a summary of examples of *in vitro* assays based on GR for various target analytes.

**Fig. 9:**
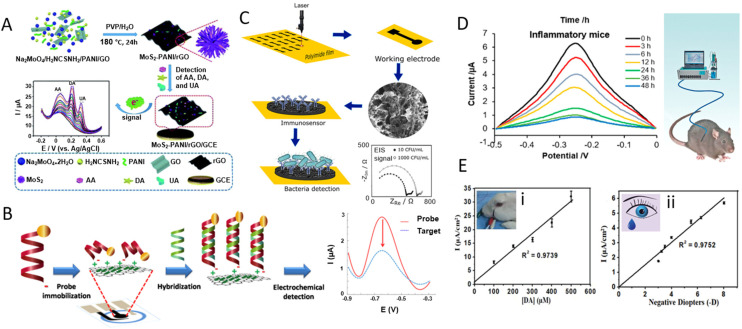
Graphene-based devices for *in vitro* and *in vivo* detection of biomarkers. [A] Schematic diagram of a MoS_2_-PANI/rGO-based electrochemical biosensor for simultaneous detection of ascorbic acid (AA), dopamine (DA), and uric acid (UA). Reprinted with permission from ref. 229. Copyright 2019 The Royal Society of Chemistry. [B] Schematic illustration of immobilization and hybridization steps of a paper-based electrochemical DNA biosensor. Reprinted with permission from ref. 235. Copyright 2016 Elsevier B.V. [C] Preparation, functionalization, and sensing scheme of a LIG-based immunosensor for bacterial detection. Reprinted with permission from ref. 232. Copyright 2020 American Chemical Society. [D] Aptamer–(Ru) probes based on graphene oxide (GO) relate inflammation duration to IFN-γ concentration in enteritis mice, advancing *in vivo* sensing. Reprinted with permission from ref. 236. Copyright 2018 American Chemical Society. [E] An *in vivo* corneal biosensor responds to varying dopamine levels in (i) rabbit's eye and (ii) human tear, opening avenues for real-time biological monitoring. Used with permission from ref. 237. Copyright 2020 Wiley.

GR-based devices have shown great success in DA detection. An abnormal level of DA in the brain may indicate Parkinson's disease and schizophrenia, among other neurological conditions.^234^ Li *et al.* prepared a 3D nanostructured composite of the MoS_2_ nanospheres and polyaniline on the rGO framework, using a one-pot hydrothermal approach.^229^ The MoS_2_-PANI/rGO suspension was then dropped on a GCE and dried to make the WE. The sensor exhibits high sensitivity for the simultaneous detection of AA, DA, and UA with three distinguishable oxidation peaks (*E*_AA peak_ = 20 mV, Δ*E*_DA peak_ = 196 mV, Δ*E*_UA peak_ = 320 mV) in DPV measurements. The responses toward AA, DA, and UA were in the linear ranges of 0.05–8.0 mM, 5–500 μM, and 1–500 μM, respectively, with LOD values of 22.20, 0.70, and 0.36 μM (Fig. 9A). Human serum and urine samples were also tested to show the sensor's selectivity and stability.

In a work by Jain *et al.*, they developed an electrochemical sensor using the AuNPs/N_2_-doped graphene nanosheet/FTO electrode. The sensor was used to detect the level of glycated hemoglobin (HbA1c) and was accordingly examined to check for diabetes mellitus. GR nanosheets were prepared using the modified Hummers’ method and then doped with nitrogen and gold NPs by solvothermal methods. The mixture was drop cast onto the cleaned FTO electrode to make the working electrode to measure the blood samples in buffer using the CV technique.^238^

Weng *et al.* reported a GFET device integrated with clustered regularly interspaced short palindromic repeats (CRISPR) assay. The sensor was used to detect both ssDNA and double-stranded DNA (dsDNA) targets. The LOD was 1 aM for the ssDNA human papillomavirus 16 synthetic target and 10 aM for the dsDNA *Escherichia coli* (*E. coli*) plasmid target without preamplification. Each chip contained a 48 GFET array to reduce the effects of measurement outliers from devices.^239^ In another work, Teengam *et al.* developed a paper-based electrochemical sensor for the detection of the human papillomavirus. The graphene–polyaniline composite was inkjet printed on paper and then functionalized with anthraquinone-labelled pyrrolidinyl peptide nucleic acid.^235^ Probe immobilization on the WE was first characterized by the EIS technique, and then the probe was investigated using SWV before and after hybridization with the target DNA, resulting in an LOD of 2.3 nM (Fig. 9B). The sensor was used to monitor the amount of HPV-DNA type 16 to identify the primary stages of cervical cancer.

Soares *et al.* reported label-free immunosensors based on the LIG to electrochemically quantify the food-borne pathogen *Salmonella enterica* serovar Typhimurium.^232^ LIG was functionalized with polyclonal anti-*Salmonella* using *N*-hydroxysuccinimide and 1-ethyl-3-(3-dimethylaminopropyl) carbodiimide as an activating agent. The immunosensor enabled the detection of the pathogen at 13 ± 7 CFU mL^−1^ in a complex medium (chicken broth), with a response time of 22 min *via* the EIS method (Fig. 9C). This work showed a low-cost preparation and fast detection process compared to CVD-graphene devices. In a work by Tan *et al.*, a GFET was functionalized with phage tail spike proteins to measure *E. coli* concentration, achieving a Dirac point shift of 35 mV when about 50 bacteria were bound to GR.^240^

GR has also been used to develop *in vitro* sensors for detecting viral infections, such as SARS-CoV-2. Virus sensing is usually achieved using impedimetric methods or GFETs. Gao *et al.* developed a GFET-based sensor with a poly-l-lysine functionalized channel to specifically detect breast cancer miRNA and SARS-CoV-2 RNA within 20 min. The LOD was as low as 1 fM using 2 μL samples.^241^ Walters *et al.* reported an Au nanoparticle-functionalized graphene resistor sensor to detect the hepatitis C virus core antigen (HCVcAg) in real time.^242^ The sensor demonstrated good sensitivity to HCVcAg of higher than 100 pg mL^−1^. In another work, LIG was functionalized with pyrenebutyric acid (PBA) as a linker to immobilize antibodies to specifically detect biomarkers including SARS-CoV-2 nucleocapsid protein (NP), immunoglobulins (Igs) against SARS-CoV-2 spike protein and C-reactive protein. The authors noted that since the SARS-CoV-2 NP structure was 90% similar to SARS-CoV NP, high interference was observed.^166^

### *In vivo* monitoring and real-time study of biological cells

In this part, we discuss how GR-based biosensors have been applied to study living organisms. The real-time monitoring capability is critical for immediate medical interventions, serving as a cornerstone for advancing personalized healthcare. By providing insights into individual health dynamics, GR-based biosensors offer the potential to tailor medical interventions and treatments to the specific needs and conditions of patients. GR is well suited for this application owing to its biocompatibility, chemical inertness, and feasibility of various functionalization routes.

In this context, Jin *et al.* developed a biosensor to monitor hydrogen peroxide (H_2_O_2_, a crucial oxidative stress and inflammation biomarker) in real time and *in vivo*.^243^ A rGO/platinum nanoparticle nanohybrid-based microneedle array was applied to facilitate seamless skin penetration, resulting in highly sensitive detection. The real-time *in vivo* testing performed on mice demonstrated the potential for this biosensor to provide continuous, non-invasive H_2_O_2_ monitoring in humans, representing an important step forward in chronic disease management. In another study, Cao *et al.* introduced an aptamer-based biosensor employing GO for signal amplification (Fig. 9D).^236^ This sensor was specifically targeted to detect interferon-γ (IFN-γ), a crucial biomarker in immune responses. The sensor achieved a low LOD of 1.3 pg mL^−1^, within the physiological range of IFN-γ in human blood. It demonstrated an exceptional ability to accurately detect IFN-γ over a period of 48 h, with no requirement for physical barriers or active drift correction algorithms, making it a reliable detection methodology.^236^

Taking a different approach, Zhang *et al.* developed a sensor for real-time dopamine detection in tear fluids, a potentially transformative approach for diagnosing and monitoring neurological conditions (Fig. 9E).^237^ They introduced novel functionalized sulfur-doped graphene, significantly enhancing the LOD down to 101 × 10^–9^ M. Their sensor exhibited remarkable stability with a one-month shelf life at 4 °C, retaining 85% sensitivity. The *in vivo* testing performed on New Zealand white rabbits confirmed its potential and suitability for tear fluid-based dopamine detection.^237^

Similarly, Wu *et al.* fabricated a microtransistor probe using GR-tethered dopamine-specific aptamers for *in vivo* dopamine monitoring in brain.^244^ With a LOD of 10 pM in artificial cerebrospinal fluid, the biosensor can detect physiologically relevant dopamine levels. *Ex vivo* and *in vivo* tests were conducted on mice to validate its potential for real time neurotransmitter tracking, which is a critical asset for understanding and managing neurological disorders.^244^

In addition to studies with animals, bacterial cells have also been directly investigated using GR-based devices. For example, Zhou *et al.* introduced a flexible MoS_*x*_/LIG-based electrochemical sensor for real-time monitoring of phenazine produced by *Pseudomonas aeruginosa* bacteria. In this work, different concentrations (100 nM – 100 μM) of pyocyanin (PYO) and phenazine-1-carboxylic acid (PCA) for the production of phenazine molecules were detected *via* the SWV method. For detecting PYO and PCA, LIG was functionalized with MoS_*x*_ on the WE *via* electrodeposition. The LIG/MoS_*x*_ electrode, with a deposition time of 60 min, showed an LOD of 0.19 μM and a sensitivity of 0.97 μA μM^−1^ for PYO in brain heart infusion (BHI). Additionally, for PCA, the LOD was reported to be 1.2 μM in BHI. Also, real-time monitoring of phenazines produced by *P. aeruginosa* in wound simulating medium showed that LIG/MoS_*x*_ could detect PYO with a LOD of 1.3 μM.^245^

### Wearable devices

Wearable devices are extremely useful for personalized health monitoring by continuously collecting and analyzing physiological signals from patients. GR is well suited for these applications due to its mechanical properties, allowing the sensors to be flexible and stretchable. It is envisioned that in the future, non-invasive wearable devices will be an affordable and convenient alternative to conventional bulky and expensive instruments.

**Fig. 10:**
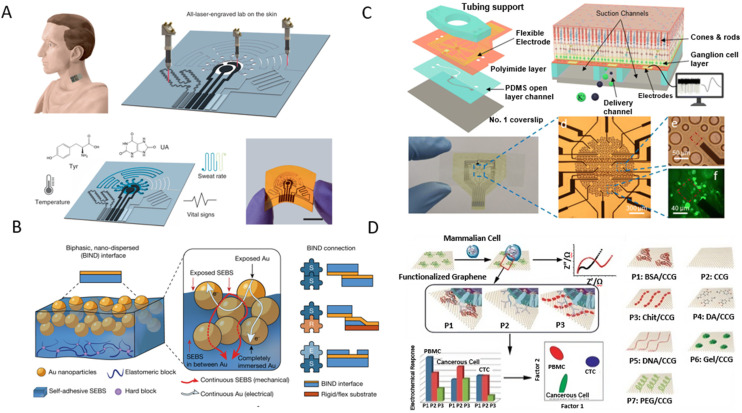
Graphene electroanalytical devices used for wearable and *ex vivo* applications. [A] An entirely laser-engraved sensing system for detection of tyrosine (TYR) and uric acid (UA) in sweat. Reproduced with permission from ref. 248. Copyright 2020 Springer Nature Limited. [B] Schematic showing that stretchable hybrid devices are typically assembled from three elementary modules (encapsulation and soft and rigid modules) and illustration of a BIND interface. Reproduced from with permission from ref. 249. Copyright 2023 Springer Nature Limited. [C] THY1.2-YFP retina analysis using graphene-based microelectrode array integrated with an Olympus IX-81 microscope for detailed retinal studies, employing advanced temperature and pressure control, high-frequency voltage sampling, and complex data processing for comprehensive anatomical and electrophysiological insights. Reprinted with permission from ref. 250. Copyright 2023 The Royal Society of Chemistry. [D] A graphene-based sensor where unique electrochemical signatures of different cell types, identified through impedance measurements and linear discriminant analysis, reveal distinct cellular properties. Reprinted with permission from ref. 251. Copyright 2017 NPG Asia Materials.

In 2016, Lee *et al.* developed a GR–gold hybrid electrochemical device for sweat-based diabetes monitoring and therapy.^246^ The soft substrate enables conformal contact with human skin under deformation, enabling stable sensing with body movements. The glucose sensor measured the electrochemical signal of the Prussian Blue functionalized GR by the reduction of H_2_O_2_ generated from glucose oxidase. Kwon *et al.* reported an all-printed wireless biosensor using biocompatible GR ink for electromyogram (EMG) recording.^247^ The high-aspect-ratio GR material offers excellent conformal lamination on human skin for EMG recording. The EMG data of specific muscle motion during flexion of each digit were recorded and analysed with a deep-learning algorithm to enable real-time classification of individual digit movement for robotic hand control. Yang *et al.* showed a wearable sensor using LIG as the multiplexing electrode to detect UA and tyrosine (TYR) from sweat as well as body temperature and the respiration rate (Fig. 10A).^248^ Laser engraving was used to fabricate the LIG electrode and pattern the double-sided medical adhesives. Microfluidic channels improved the rate of collecting sweat from the skin. Sensor validation was performed by applying the integrated system on different body parts and measuring the UA and TYR levels in real time. The results showed its reliability for the non-invasive monitoring of physiological signals and the potential for disease diagnosis such as hyperuricemia.

Cai *et al.* took advantage of the biocompatibility and flexibility of graphene fiber fabric, modified with glucose oxidase and chitosan, to fabricate a wearable glucose monitoring patch.^252^ The sensor was able to detect glucose concentrations from 2 μM to 650 μM, aligned with the human interstitial fluid's physiological glucose range. They carried out tests to compare the sensor output to traditional finger-prick blood samples, showing a strong correlation between them, confirming its efficacy and potential for continuous, non-invasive glucose monitoring.^252^ Recently, Kireev *et al.* developed GR bioimpedance tattoos using CVD grown GR for continuous monitoring of arterial blood pressure.^253^ The atomically thin, self-adhesive, light weight, and unobtrusive GR electronic tattoos as human bioelectronic interfaces enabled a longer monitoring time than that previously reported. Combined with the cycling-trained machine learning regression model, the accuracies were 0.06 ± 2.5 mm Hg (diastolic) and 0.2 ± 3.6 mm Hg (systolic).

It is worth highlighting that on-body measurements need good interconnects between soft tissue and Si-based devices. Stress at the interface may result in debonding failure. In addition, the encapsulation of devices is also essential since any contact on the interface can result biological fouling and device failure. To overcome these issues, Jiang *et al.* developed a universal interface that connected soft, rigid, and encapsulation modules through simply pressing (Fig. 10B). The connection between soft modules shows 3× electrical stretchability and 10× mechanical stretchability compared to commercial pastes. A stretchable device was also assembled using these connection modules for tests as a proof of concept.^249^

### *Ex vivo* studies

GR-based electroanalytical devices are being increasingly employed in *ex vivo* studies, which enable the testing of drug effects outside a living organism (such as in organ-on-chip devices), often in a controlled laboratory environment.^254,255^ Such assays are critical for drug screening, especially in understanding drug interactions with target cells like cancer cells or neurons. The exceptional electronic properties of graphene enable sensitive detection and quantification of cellular responses, providing crucial insights into drug efficacy and potential side effects. Moreover, the integration of sensors that mimic the microenvironment of human organs has paved the way for more realistic and high-throughput drug testing. These advances highlight the transformative potential of GR-based devices in accelerating drug discovery and improving patient-specific therapeutic strategies.^256^

Recent research has advanced the application of GR-based microelectrode array (MEA) platforms for *ex vivo* analysis.^250^ In this work, the GR-based MEAs, paired with an Olympus IX-81 inverted epifluorescence microscope, operated in oxygenated media at a temperature of 36 °C. The use of two syringe pumps created a controllable negative pressure. The readout methodology from these GR electrodes utilized a 16-channel amplifier and a lab-made printed circuit board zero-insertion-force connector, allowing for the recording of voltage at a 20 kHz sampling rate. Data processing incorporated spike sorting with the Plexon offline sorter and a Butterworth filter for further analysis. There has been considerable progress in detecting THY1.2-YFP retinas using GR-based MEAs, which is instrumental in creating a detailed anatomical and electrophysiological analysis (Fig. 10C).

In another work, Wu *et al.* used a modified Hummers’ method of preparing GR to develop an array-based system for cell sensing using a chemical nose/tongue approach that exploits subtle changes in the physicochemical nature of different cell surfaces.^251^ The chitosan-mediated graphene suspension was added dropwise onto the pretreated GCE and the EIS signals were recorded. The interaction of the cells with the functionalized GR array depended on the surface properties of the cell. Each type of cell had its colour-change fingerprint. To deconvolute the electrochemical reactions that provided discriminating signatures, the electrochemical data set was classified for all seven GR probes using linear discriminant analysis (Fig. 10D).

**Fig. 11:**
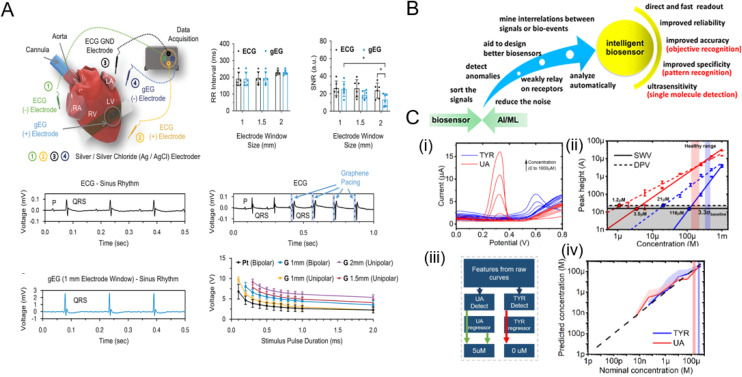
*Ex vivo* studies and emerging fields based on graphene-based biodevices. [A] *Ex vivo* cardiac electrophysiology sensing and pacing with GFETs. Reprinted with permission from ref. 257. Copyright 2023 Wiley-VCH GmbH. [B] Schematic highlighting the benefits of using machine learning (ML) in conjunction with biosensors. Adapted with permission from ref. 258. Copyright 2014 American Chemical Society. [C] Enhancement in limit of detection (LOD) using data processing techniques: (i) Raw DPV curves showing peaks for tyrosine (TYR) and uric acid (UA), (ii) extracted LODs using single peak analysis, (iii) the ML architecture that was developed to boost LOD by at least two orders of magnitude compared to conventional voltammetry methods (iv). Adapted with permission from ref. 259. Copyright 2022 Elsevier.

Lin *et al.* leveraged electronic GR tattoos as soft, tissue-imperceptible, and transparent bioelectronic interfaces.^257^ They meticulously characterized GFETs and employed them for monitoring cardiac activity in the well-established *ex vivo* Lutgendorf-perfused mouse heart model. In this setting, the electrogram recorded by graphene (gEG) was contrasted with the traditional far-field ECG, captured simultaneously using commercial Ag/AgCl needle electrodes in the perfusion bath. Notably, when comparing the signal-to-noise ratio (SNR) across varying gEG electrode sizes, the 1 mm electrode was found to be very effective, consistent with earlier observations. The capability of these GFET electrodes in cardiac actuation was also explored, highlighting their performance in both unipolar and bipolar modes. A pivotal pacing strength–duration curve, essential for understanding the efficiency of various GFET electrodes was studied, positioning the 1 mm unipolar GFET electrode favourably against a reference platinum electrode (Fig. 11A).

### Emerging topics

In recent years, there has been growing interest in the incorporation of machine learning (ML) algorithms into electrochemical biosensors (Fig. 11B). The recent trend in electrochemical sensor research has been to apply complex potential waveforms to the sensor in order to extract more information than just a linear potential sweep as in CV or pulse voltammetry (such as SWV, DPV).^260^ These complex waveforms may consist of using sinusoid superimposed ramps that form large amplitude alternating current voltammetry (LAACV),^261^ semicircular sweep voltammetry that employs a varying scan rate,^262^ or designer sinusoidal waveforms.^263^ Conventionally, data processing in the form of manual baseline subtraction and single peak-based trend analysis is used to quantify and detect analytes. One approach for data processing is to use ML to learn the subtle and complex signals associated with sensing.^258^ One of the key advantages of using ML in electrochemical biosensors is to facilitate pattern recognition and multiplex correlative data processing of the complex sensor output signals. It is common for other analytical/diagnostic methods such as spectroscopy or imaging techniques to use ML; however, the use of ML in electroanalytical biosensors has been slow to implement due to the lack of data corpus and complex signal variations in biological media.^258^ Using CV, ML-based support vector machine (SVM) models have been used to estimate the nitrate concentration.^264^ In other works,^265,266^ fast scan cyclic voltammetry (FSCV) was used along with ML models to selectively and accurately predict the neurotransmitter levels.

The power of ML is in the vast data processing it can handle along with the added advantages of combining multiple sources of data. Multiple electrochemical methods, such as CV, SWV, DPV, and LAACV, are known to have varying sensitivities^267^ and varying background current rejection,^268^ and hence are expected to behave differently with different sensor materials, analytes, and media. Kammarchedu *et al.* demonstrated the effect of the multimodal effects and data fusion techniques and their effects on the sensing performance of the sensors by improving the LOD of TYR and UA in artificial saliva on LIG-based sensors 100 times by using ML (Fig. 11C).^259^

Furthermore, ML can also be used to predict new materials and material modifications for optimizing the sensing performance.^269^ One of the limiting factors for rapid sensor development is the slow process of material synthesis and subsequent benchmarking to find ideal material–biomolecule pairs. Towards this goal, density functional theory (DFT) studies as well as the establishment of other theoretical frameworks are crucial. One way to do this is to couple experimental and theoretical results in published studies in order to build a database that can be used to train an ML model.^270^

## Discussion and conclusion

The remarkable properties of GR, precise synthesis techniques, and its diverse applications in biosensing and bioelectronics present a promising avenue for addressing the evolving challenges in the healthcare landscape. GR can be synthesized at scale using various top-down and bottom-up techniques. Furthermore, GR properties can be altered/tuned using doping, molecular/chemical functionalization, defect engineering, *etc*. Electrical, electrochemical, and electro-optical transduction methods can be utilized to fabricate devices on both rigid and flexible substrates. These GR-based electroanalytical devices offer the potential to revolutionize disease diagnosis, personalized medicine, and remote patient monitoring, particularly in the context of chronic diseases and infectious outbreaks, as well as bioelectronics and life science research when interfacing with biological cells. GR's exceptional electronic attributes augment the sensitive detection of cellular responses, thus enriching our understanding of drug efficacies and probable adverse effects. Recent innovations, including GR-based microelectrode array platforms and integrations that mirror human organ microenvironments, have markedly enhanced the realism and throughput of drug testing. These advancements signify a transformative era, potentially catalysing drug discovery and refining patient-tailored therapeutic strategies.

Although significant progress has been made in the past years in GR-based wearable devices, there are still bottlenecks, such as power management, system integration, and commercializing high-performance sensors.^255^ In addition, achieving reproducibility in synthesis to maintain GR's properties during functionalization and ensuring biocompatibility for *in vivo* applications, demand careful consideration. Furthermore, the issue of sensor calibration and drift add to the challenges of prolonged storage and usage of these electroanalytical devices. Several drift correction algorithms have been developed^271–273^ to offset sensor drift and calibration-free sensors have been proposed^274,275^ to mitigate the need for recalibration. Still, further research and novel techniques are needed to solve these problems. Lack of unified standards for synthesis and fabrication of biosensor materials and devices, including those based on graphene, is also another major challenge which needs to be addressed for utilization of GR-based biosensors in the healthcare market. Overcoming these hurdles is crucial to realizing the full transformative potential of GR-based biodevices in public health and life sciences.

In the face of these challenges, this review article underscores the transformative role that GR-based devices can play in addressing critical healthcare needs. It is envisioned that smart devices enabling continuous and non-invasive monitoring of human health could be combined with machine learning and the Internet of Things (IoT), to generate predictive algorithms for early disease diagnosis. Especially, emerging technologies such as machine learning can help in enhancing the sensing performance of sensors as well as predict novel materials for sensing applications. As technology continues to advance, the integration of these devices with digital platforms holds the promise of ushering in an era of preventive and precision medicine, ultimately improving patient outcomes and healthcare management on a global scale.

## Conflicts of interest

There are no conflicts to declare.
